# Karyological investigations and new chromosome number reports in *Bellevalia* Lapeyrouse, 1808 and *Muscari* Miller, 1758 (Asparagaceae) from Algeria

**DOI:** 10.3897/CompCytogen.v10i1.6445

**Published:** 2016-03-21

**Authors:** Nadjat Azizi, Rachid Amirouche, Nabila Amirouche

**Affiliations:** 1 University of Sciences and Technology Houari Boumediene, Faculty of Biological Sciences, LBPO lab., Team: Biosystematics, Genetic and Evolution. USTHB, PO box 32 El-Alia, Bab-Ezzouar, 16110 Algiers, Algeria

**Keywords:** Algeria, *Bellevalia*, *Muscari*, chromosome number, karyotype, polyploidy

## Abstract

Karyological investigations were carried out on four species of *Bellevalia* Lapeyrouse, 1808 and *Muscari* Miller, 1758 (Asparagaceae) sampled in contrasting bioclimatic conditions of Algeria. The endemic *Bellevalia mauritanica* Pomel, 1874 was found to have a tetraploid cytotype 2n = 4x = 16 and an octoploid 2n = 8x = 32 which is a new report. The chromosome number 2n = 2x = 18 in *Muscari comosum* (Linnaeus, 1753) Miller, 1768 and *Muscari maritimum* Desfontaines, 1798 was in conformity with earlier reports. The latter species reveals a lesser bimodality of the karyotype. Within *Muscari neglectum* Gussone ex Tenore, 1842 pentaploid (2n = 5x = 45), hexaploid (2n = 6x = 54) and very rare octoploid cytotype (2n = 8x = 72) have been reported in Algeria. Principal component analysis performed on basis of karyotype parameters, showed a segregation of the different cytotypes. This study provides new karyological information, which is discussed in a taxonomic context.

## Introduction

The Hyacinthaceae is one of the most important families of Asparagales, containing about 70 genera and 700-1000 species (Speta 1998, Pfosser and Speta 1999, Ali et al. 2012). Currently, they are included in the expanded Asparagaceae *sensu* APGIII (2009) as subfamily Scilloideae comprising four tribes Hyacintheae, Ornithogaleae, Urgineeae and Oziroëeae (Chase et al. 2009). Except Oziroëeae, they show a disjunct distribution pattern between the Mediterranean area, north-west Africa, western Asia and sub-Saharan Africa (Sanmartin et al. 2010, Ali et al. 2012, Pfosser et al. 2012). Hyacintheae is undoubtedly the most significant tribe, according to the number of species. Many of them present interesting patterns for examining karyological evolution and polyploidy in relation with their geographical distribution (Speta 1998, Stedje 2001, Hamouche et al. 2010, Goldblatt et al. 2012, Weiss-Schneeweiss and Schneeweiss 2013). Actually, many new chromosome records have lead to description of new species and/or change in taxonomic and nomenclatural status. That is the case of *Bellevalia* Lapeyrouse, 1808 and *Muscari* Miller, 1758.

These genera display similarities in many morphological traits, particularly concerning the floral bud stage and mature fruits. On the basis of morphological criteria, they have been traditionally linked together (Garbari and Greuter 1970) and their close relationship was supported by molecular phylogeny, placing them in the same clade (Pfosser and Speta 1999). Moreover, the geographical range of both genera covers the same areas from the western Mediterranean region (Morocco, Algeria) eastwards throughout Europe and southwestern Asia (Johnson 2003, Nersesian 2001, Bareka et al. 2008, Jafari et al. 2008, Jafari 2012a, 2012b, Borzatti Von Loewenstern et al. 2013, Demirci et al. 2013). However, from the karyological point of view, *Bellevalia* and *Muscari*, differ significantly from each other. The genus *Bellevalia* has a low basic chromosome number x = 4 with large chromosomes and several ploidy levels from 2x to 8x (Speta 1998, Johnson 2003, Yaylaci et al. 2009), while the genus *Muscari* is characterized by the base chromosome number x = 9, with more bimodal karyotype (Garbari 1984, Bentzer and Ellmer 1975, Ruiz Rejón and Oliver 1981).

Within the genus *Bellevalia*, endemic species have been recently discovered, mainly in Anatolia. Some of these new described species are diploids (2n = 2x = 8), such as *Bellevalia leucantha* K. Persson, 2006, *Bellevalia malatyaensis* Uzunhisarcikli & Duman, 2013 and *Bellevalia koyuncui* Karabacak & Yildirim, 2015 (Persson 2006, Uzunhisarcikli et al. 2013, Karabacak et al. 2015). Polyploid species such as *Bellevalia pseudolongipes* Karabacak & Yildirim, 2014 (Karabacak et al. 2014), *Bellevalia clusiana* Grisebach, 1844 (Yaylaci et al. 2009) and *Bellevalia edirnensis* N.Özhatay & Mathew, 1991 (Özhatay et al. 1991b) were identified as triploid, tetraploid and hexaploid respectively. Recently, a new hexaploid species, *Bellevalia juliana* Bareka, Turland & Kamari, 2015 (Bareka et al. 2015) was found in Greece. In Tunisia, two tetraploid endemic species were described, *Bellevalia galitensis* Bocchieri & Mossa, 1991 and *Bellevalia dolichophylla* Brullo & Minissale, 1997 (Bocchieri and Mossa 1991, Brullo and Minissale 1997). New populations of these species were recently recorded by Troìa et al. (2014). According to Brullo et al. (2009), the Tunisian species show a close relationship with *Bellevalia pelagica* C.Brullo, Brullo & Pasta, 2009 also tetraploid, and endemic to Lampione islet (Sicily). Cytogenetic studies (Bareka et al. 2008, 2012) and phylogenetic analysis (Borzatti Von Loewenstern et al. 2013), performed on populations occurring in Greece and Italy respectively, highlighted the diversity in *Bellevalia* and raised questions about the taxonomic relationships and the origin of polyploids.

The situation in the genus *Muscari* is more complex both taxonomically and karyologically. Within this genus, four groups were traditionally recognized, alternatively considered as sections, subgenera or as separate genera (Maire 1958; Garbari and Greuter 1970; Davis and Stuart 1980; Speta 1998; Jafari and Maassoumi 2011): *Leopoldia* Parlatore, 1845, *Muscarimia* Kosteletzky ex A.S. Losina-Losinskaja, 1935, *Pseudomuscari* Garbari & Greuter, 1970 and *Muscari* Miller, 1754 (= *Botryanthus* Kunth, 1843). Species belonging to the subgenus *Leopoldia* are principally diploid although few triploid and tetraploid cytotypes have been quoted (Ruiz Rejón et al. 1985; Nersesian 2001). Species of this group, were also discovered mainly in Iran such as *Leopoldia ghouschtchiensis* Jafari & Maassoumi, 2011, *Leopoldia tabriziana* Jafari, 2012 and *Leopoldia tijtijensis* Jafari, 2012 (Jafari and Maassoumi 2011, Jafari 2012a, 2012b). In Turkey, a new endemic species, *Muscari erdalii* N.Özhatay & S.Demirci, 2013 (Demirci et al. 2013) was identified. However, within the subgenus *Muscari*, the occurrence of polyploidy is higher, particularly among the polymorphic complex *Muscari neglectum* Gussone ex Tenore, 1842. Populations occurring in Greece and Turkey display a ploidy series ranging from 2x to 8x (Karlén 1984, Garbari 2003). In the Iberian Peninsula, populations of *Muscari neglectum*, reported as tetraploid, pentaploid and hexaploid, were treated by Suárez-Santiago et al. (2007) as separate species according to their ploidy level.

Despite its biogeographical position in the south-western Mediterranean area, Algeria suffers from an obvious lack of cytotaxonomic data (Amirouche and Misset 2009). This is why it is necessary to start our research by karyological investigations. According to the ancient floras of Algeria (Desfontaines 1798-1799, Battandier and Trabut 1895-1902, Maire 1958, Quézel and Santa 1962), *Bellevalia* and *Muscari* comprise four and five species respectively. This paper is part of an ongoing program on Asparagales in Algeria and aims to complete chromosomal counts, karyotypes knowledge and geographical distribution of the polyploidy. It focuses on the endemic *Bellevalia mauritanica* Pomel, 1874, and *Muscari comosum* (Linnaeus, 1753) Miller, 1768, *Muscari maritimum* Desfontaines, 1798 and *Muscari neglectum* Gussone ex Tenore, 1842.

## Material and methods

### Sampling and taxonomic determinations

Populations used in this study were sampled from March to May 2010–2012 in various ecogeographic areas of Northern Algeria (Table 1). In each site, 5–10 bulbs were collected and cultivated in the experimental garden of Houari Boumediene University of Sciences and Technology (Algiers). Taxonomic determinations were made based on several North-Africa and Mediterranean Floras: Desfontaines (1798–1799), Battandier and Trabut (1895–1902), Maire (1958), Quézel and Santa (1962) and Davis and Stuart (1980). The specialized taxonomic and nomenclatural websites, the *African Plant Database* (Dobignard and Chatelain 2010–2013) and the *World Check List of Selected Plants* (Govaerts 2015) were also consulted.

**Table 1.** Origin of the studied species and geographical information of the sampling sites.

Taxon *	Locality/site	Biogeo. Sect.	Lat.	Long.	Alt.
*Bellevalia mauritanica* Pomel	Constantine, Tiddis	C1	36°29'N	06°30'E	546
	Mostaganem, Stidia	O1	35°47'N	00°05'W	35
	Miliana, Ain Torki	A1	36°20'N	02°18'E	715
	Algiers, Ouled Fayet	A1	36°44'N	02°57'E	186
*Muscari comosum* (L.) Miller	Tipaza, Ain Taghourait	A1	36°35'N	02°37'E	219
	Chlef, Ténès	A1	36°19'N	01°14'E	210
	Tizi Ouzou, Zekri	K1	36°46'N	04°34'E	800
*Muscari maritimum* Desfontaines	Djelfa, Guelt es Stel	H1	35°09'N	03°01'E	907
*Muscari neglectum* Gussone ex Tenore	Constantine, Ain El Bey	C1	36°18'N	06°36'E	750
	Constantine, Tiddis	C1	36°29'N	06°30'E	546
	Sétif, Djemila	C1	36°12'N	04°22'E	459
	Tlemcen, Mansourah	O3	34°51'N	01°18'W	1038

* Nomenclature according to Maire (1958), Dobignard and Chatelain (2013), Govaerts (2015).

Biogeographical sectors are from Quézel and Santa (1962): A: Algiers, C: Constantine, K: Kabylie, O: Oran, H: Hodna (High Plains).

Lat. Latitude , Long. Longitude , Alt. Altitude in meters.

### Chromosome preparations

Mitotic preparations were performed on young root-tips obtained from potted plants. The chromosome observations were performed using the standard Feulgen technique for staining tissues (Jahier et al. 1992), with little modifications. Root-tips were pretreated in 8-hydroxyquinoline (0.002%) or in 0.25 % aqueous colchicine for 5 hours at room temperature, then fixed in Carnoy fixative solution (3 : 1 (v/v) ethanol : acetic acid) at 4°C for at least 48 hours. Hydrolysis was made in 1N HCl for 7–9 min at 60°C before staining with the usual Schiff reagent. Root-tips were squashed in a drop of 45% acetic acid. The observations were made using a Carl Zeiss Axiostar-Plus microscope equipped with a Canon digital camera.

### Karyotype and idiogram constructions

Measurements for karyotype and idiogram constructions were based on at least five well-spread metaphase plates of different individuals. The arrangement of homologous pairs was made using MICROMEASURE Software version 3.3 (Reeves 2001). Chromosomes are described according to the nomenclature of Levan et al. (1964) based on the chromosomal arm ratio (r = long arm/short arm) and the centromeric index (CI % = short arm/long arm + short arm × 100): metacentric (m), submetacentric (sm), subtelocentric (st) and telocentric (t). Karyotype asymmetry indices were estimated following the proposal of Peruzzi and Eroğlu (2013). The intrachromosomal asymmetry index is represented by the mean centromeric asymmetry M_CA_ = A × 100, where A is the average ratio of long arm-short arm/long arm + short arm, according to Watanabe et al. (1999). The interchromosomal asymmetry index is the coefficient of variation of chromosome length CV_CL_ = A_2_ × 100 (Paszko 2006) where A_2_ is the standard deviation of chromosome length/mean chromosome length (Romero Zarco 1986). The coefficient of variation of the centromeric index CV_CI_ = S_CI_/X_CI_ × 100 is the ratio between the standard deviation S_CI_ and the mean centromeric index X_CI_ (Paszko 2006).

### Multivariate analysis

In order to compare the karyotypes of the studied species, a Principal Component Analysis (PCA) was performed using STATISTICA Software version 6. Analysis was based on six fundamental karyological parameters as proposed by Peruzzi and Altinordu (2014): chromosome number (2n), chromosome base number (x), total haploid chromosome length (THL), mean centromeric asymmetry MCA, coefficient of variation of chromosome length CVCL and coefficient of variation of the centromeric index CVCI.

## Results

**Figure 1.**
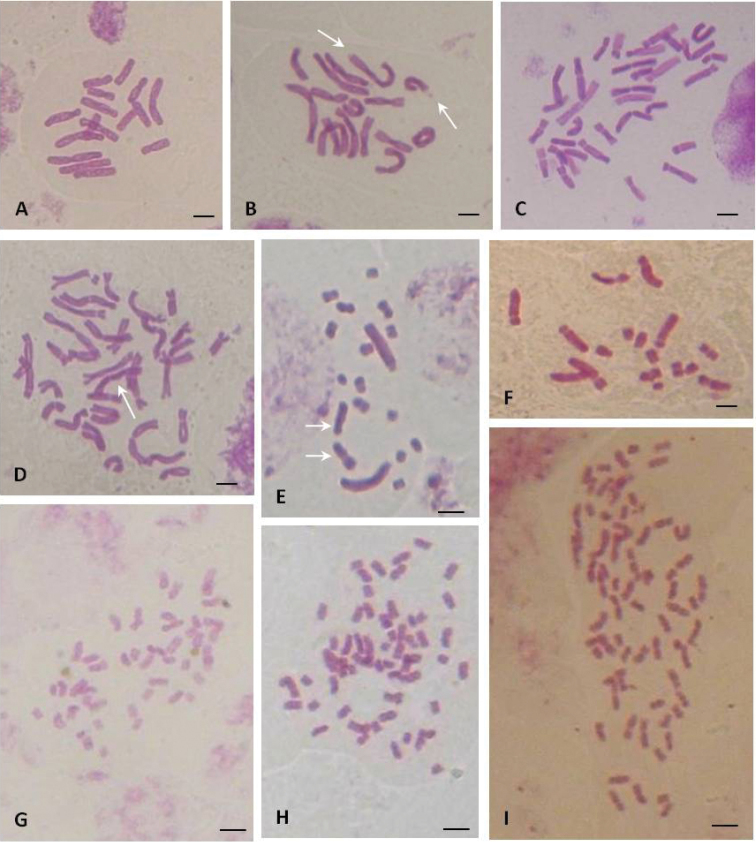
Mitotic metaphases of *Bellevalia* and *Muscari* from Algeria. **A–D** *Bellevalia mauritanica*: A 2n = 16 (Tiddis) **B** 2n = 16 (Stidia) arrows indicate satellites **C** 2n = 32 (Ouled Fayet) **D** 2n = 32 (Ain Torki) arrow indicates a supernumerary chromosome **E** *Muscari comosum* 2n = 18 (arrows: 2^st^ polymorphic pair) **F** *Muscari maritimum* 2n = 18 (Guelt es stel) **G–I** *Muscari neglectum*: **G** 2n = 45 (Ain El Bey) **H** 2n = 54 (Tiddis) **I** 2n = 72 (Djemila). Scale bars = 5 µm.

**Figure 2.**
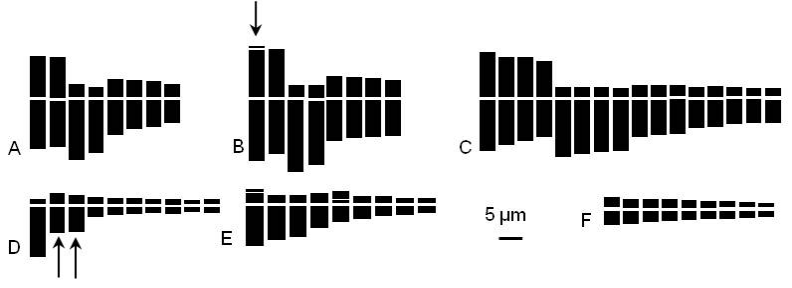
Idiograms of the four studied species of *Bellevalia* and *Muscari*. **A** *Bellevalia mauritanica* 4x (Tiddis) **B** *Bellevalia mauritanica* 4x (Stidia) arrow indicates satellite **C** *Bellevalia mauritanica* 8x **D** *Muscari comosum* 2x (Arrows indicate 2^st^ polymorphic pair) **E** *Muscari maritimum* 2x **F** *Muscari neglectum*: symbolized haploid set for 5x, 6x and 8x.

Chromosome numbers, ploidy level and karyotype characteristics of the four studied species of *Bellevalia* and *Muscari* occurring in Algeria are summarized in Tables 2–3. Representative metaphases and the idiograms are shown in Figs 1–2.

**Table 2.** Characteristics of karyotype structure in cytotypes of *Bellevalia* and *Muscari*.

Taxon/ Cytotype/Pop.	MCL (µm) ± SD	CLR (µm)	THL (µm) ± SD	M_CA_	CV_CL_	CV_CI_
*Bellevalia mauritanica* 4x (Tiddis)	11.63 ± 0.70	07.00–17.10	093.05 ± 04.63	32.23	32.81	33.05
*Bellevalia mauritanica* 4x (Stidia)	14.23 ± 0.84	10.05–20.47	113.86 ± 06.06	35.43	28.23	34.80
*Bellevalia mauritanica* 8x Ouled Fayet, Ain Torki	10.71 ± 0.70	06.05–18.05	171.40 ± 08.84	42.07	35.27	42.37
*Muscari comosum* 2x Tipaza, Ténès, Zekri	03.68 ± 0.39	01.94–10.49	033.51 ± 03.22	19.97	73.8	29.55
*Muscari maritimum* 2x Guelt es Stel	05.29 ± 0.27	02.37–09.38	047.64 ± 01.53	47.19	36.97	28.09
*Muscari neglectum* 5x Ain El Bey	03.17 ± 0.25	01.99–04.73	072.96 ± 05.53	15.65	23.97	4.78
*Muscari neglectum* 6x Tiddis	03.33 ± 0.10	01.80–05.39	089.96 ± 02.2	17.94	25.94	6.61
*Muscari neglectum* 8x Djemila	03.42 ± 0.36	01.96–05.35	123.24 ± 12.72	14.86	26.18	5.84

MCL : mean chromosomal length , CLR : chromosome length range , THL : total haploid length , MCA : mean centromeric asymmetry (Peruzzi and Eroğlu 2013), CVCL : coefficient of variation of chromosome length , CVCI : coefficient of variation of centromeric index (Paszko 2006) .

**Table 3.** Chromosome number, ploidy and karyotype formula in the studied species of *Bellevalia* and *Muscari*.

Taxon	Populations	Ploidy	2*n*	Karyotype formula
*Bellevalia mauritanica*	Tiddis	4x	16	4m + 4st + 8sm
	Stidia	4x	16	4m-sat + 4st + 8sm
	Ouled Fayet, Ain Torki	8x	32	8m + 8st + 16sm
*Muscari comosum*	Tipaza, Ténès, Zekri	2x	18	2t + (1m + 1sm) + 14m
*Muscari maritimum*	Guelt es Stel	2x	18	6st-sat + 6sm-sat + 6m
*Muscari neglectum*	Ain El Bey, Mansourah	5x	45	45m
	Tiddis	6x	54	54m
	Djemila	8x	72	72m

### *Bellevalia mauritanica* Pomel, 1874

Mitotic observations showed tetraploid and octaploid cytotypes with base number x = 4. The tetraploid cytotypes 2n = 4x = 16 (Fig. 1A–B; 2A–B) was found in two populations from two contrasted biogeographical sectors. Plants from biogeographical sector of Constantine (Tiddis) grow on clayey-marly soil. Their chromosomes show a total haploid length THL = 93.05 μm with mean length per chromosome (CLR) ranging from 7.00 to 17.1 µm (Table 3). The karyotype consists of 4m + 4st + 8sm. Specimens from the biogeographical sector of Oran (Stidia) occurring on coastal sand dunes are distinguished by much larger chromosomes. The mean length per chromosome (CLR) is 10.05-20.47 µm and THL = 113.86 µm (Table 3) with a karyotype formula 4m-sat + 4st + 8sm. This karyotype is distinguished by two terminal satellites on the first largest metacentric pair (Fig. 2B). Except the occurrence of the satellites, the structure of the two karyotypes is similar regarding the centromeric asymmetry values and the coefficient of variation (Table 3).

The octoploid cytotype 2n = 8x = 32 (Fig. 1C, 2C) was found in two populations from Ouled Fayet and AinTorki of the biogeographical sector of Algiers. This cytotype is characterized by a larger THL 171.40 μm and CLR values more extensive (6.05-18.05 μm). The karyotype formula is quite similar to that of the tetraploids. One submetacentric supernumerary chromosome was occasionally observed in octoploid individuals (Fig. 1C). The centromeric asymmetry indices (M_CA_) of tetraploid and octaploid cytotype are rather different while the coefficients of variation (CV_CL_) are much closer.

### *Muscari comosum* (Linnaeus, 1753) Miller, 1768

This species is widespread in the north of Algeria. Examined populations were diploids with 2n = 18 chromosomes and a base number x = 9 (Fig. 1E). The mean length per chromosome is comprised between 1.94 µm to 10.49 µm and total length THL = 33.51 µm (Table 3). The karyotype is distinguished by two large pairs of chromosomes and seven other pairs much smaller. The first pair is telocentric; the second pair constituted by one metacentric and one submetacentric chromosome is polymorph due to structural heterozygosity (Figs 1E, 2D). All the remaining small chromosomes are metacentric. The karyotype formula is 2t + (1m + 1sm) + 14m. The values of the centromeric asymmetry (M_CA_) and the coefficient of variation (CV_CL_) are 73.8 and 19.97 respectively.

### *Muscari maritimum* Desfontaines, 1798

*Muscari maritimum* is less common. The studied population lives on the sand dunes in the steppe high plains of the Saharan border (Guelt es Stel). It is also diploid with 2n = 18 (Fig. 1F). The mean length of chromosomes is between 2.37 µm and 9.38 µm with a THL = 47.64 µm (Table 3). The karyotype is characterized by 6st-sat + 6sm-sat + 6m (Fig. 2) showing two satellites: terminal on the first subtelocentric pair, and intercalary on the fifth submetacentric pair. Compared to *Muscari comosum*, *Muscari maritimum* have a less asymmetrical karyotype reflected in a low value of its centromeric asymmetry index (M_CA_).

### *Muscari neglectum* Gussone ex Tenore, 1842

**Figure 3.**
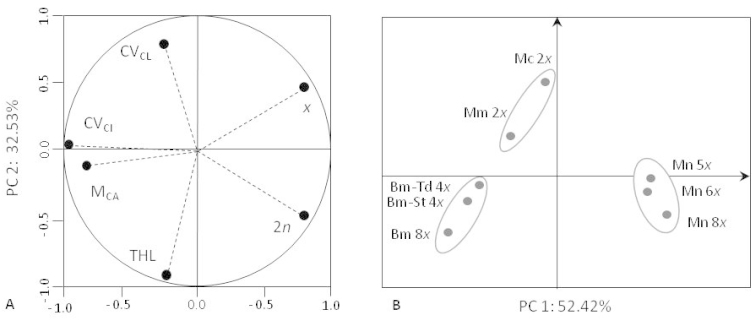
Principal Component Analysis of the eight cytotypes of *Bellevalia* and *Muscari*. **A** Correlation loadings of the six karyotype variables with PC1 and PC2 (abbreviations in Table 3) **B** Corresponding projection of the eight cytotypes: Bm *Bellevalia mauritanica* (Tiddis), *Bellevalia mauritanica* (Stidia), Mc *Muscari comosum*, Mm *Muscari maritimum*, Mn *Muscari neglectum*.

In this species, three cytotypes were observed: pentaploid 2n = 5x = 45, hexaploid 2n = 6x = 54 and octaploid 2n = 8x = 72 (Figs 1G–I). All cytotypes were encountered in the eastern biogeographical sector of Constantine (Ain El Bey, Tiddis and Djemila). The western population of Mansourah (Tlemcen) is pentaploid (Tables 1–2). Compared to the previous species, chromosomes are markedly small with mean lengths between 1.80 µm and 5.39 µm and no significant difference among the three karyotypes (Table 3). This species is characterized by a rather symmetrical karyotype comprising only metacentric chromosomes (Figs 1–3, Table 3) with total length depending on the ploidy level. The centromeric asymmetry indices (M_CA_) and the coefficients of variation are also similar.

### Karyotype relationship

In order to estimate the karyological relationship among the studied taxa, a principal component analysis (PCA) was carried out on the 8 populations, each representing different species and/or cytotypes (Fig. 3). The pattern of correlation loadings of the variables (Fig. 3A) highlights the major role of PC1 and PC2. Cumulative variance explained by these two first components approaches to 85% of the total information. The formation of PC1 was due to intrachromosomal asymmetry parameter M_CA,_ the coefficient of variation of the centromeric index CV_CI_ (negative values) and to chromosome numbers 2n and x (positive values) which have a discriminant power > 0.79 (data not shown). PC2 is well described by the variables THL and CV_CL_ (inversely correlated) and, in least degree, once again, by the chromosomal numbers 2n and x (Fig. 3A). As expected, the projection of the taxa on the first two axes confirms the divergence between the cytotypes representing the genus *Muscari* from those of genus *Bellevalia* (Fig. 3B).

Cytotypes of *Bellevalia mauritanica* constitute a clearly distinct group, in which the two tetraploid cytotypes (from Stidia and Tiddis) shows close relationship. The octoploid cytotype (2n = 8x = 32) can be discreetly distinguished probably because of a higher value of the total haploid length (THL).

The karyotypes of the studied species of *Muscari* constitute two other clusters significantly different from each other (Fig. 3B): the first cluster is limited to the positive values of PC1 and involves all the 5x, 6x and 8x cytotypes of *Muscari neglectum*; the second cluster, showing positive values of PC2, relates to diploid karyotypes of *Muscari comosum* and *Muscari maritimum*. This distribution matches the different affiliation of the species to the two subgenera *Botryanthus* and *Leopoldia* respectively. The diploid species belonging to subgenus *Leopoldia* e.g. *Muscari maritimum* [= *Leopoldia maritima* (Desfontaines, 1798) Parlatore, 1845] and *Muscari comosum* [= *Leopoldia comosa* (Linnaeus, 1753) Parlatore, 1847] are well separated due to different asymmetry chromosomal indices M_CA_, CV_CI_ and CV_CL_. Within the *Muscari neglectum* group the three ploidy levels did not show any significant differentiation.

## Discussion

### Chromosome number and polyploidy in genus *Bellevalia*

The studied populations of *Bellevalia mauritanica* display two ploidy levels, tetraploid (2n = 4x = 16) and octoploid (2n = 8x = 32). This species was previously known as exclusively tetraploid besides twelve other species of the genus (Brullo et al. 2009, Bareka et al. 2012).

Usually, in the genus *Bellevalia*, the karyotypes show satellites on either the first, the second or the third pair of chromosomes (Bothmer and Wendelbo 1981, Bareka et al. 2008, 2012). Our tetraploid *Bellevalia mauritanica* from Stidia shows a similar chromosome arrangement and bears one pair of satellites on the first metacentric pair.

The octoploid level is reported here in *Bellevalia mauritanica* for the first time The polyploidy is quite abundant in *Bellevalia*, 2x, 3x, 4x, 6x and 8x levels have already been reported (Musano and Maggini 1976, Özhatay et al. 1991a, Özhatay and Johnson 1996, Johnson 2003, Yaylaci et al. 2009, Bareka et al. 2012, Karabacak et al. 2014). The octoploid level is rare and it seems known in two species only, *Bellevalia longistyla* (Miscz.) Grossheim, 1928 (Özhatay and Johnson 1996, Johnson 2003) and *Bellevalia olivieri* (Baker) Wendelbo, 1985 (Bareka et al. 2012, 2015). Singular populations with 2n = 4x = 32 have been also quoted for *Bellevalia glauca* (Lindley) Kunth, 1843 and *Bellevalia sarmatica* (Pallas ex Miscz.) Woronow, 1927 (Bothmer and Wendelbo 1981).

In some octoploid cytotypes of *Bellevalia mauritanica*, we observed one large and metacentric supernumerary chromosome, similar to all the other homologues. It seems to be a very interesting case of aneuploidy, which has not yet been reported, to our knowledge, in genus *Bellevalia* (P. Bareka pers. comm.). Only B chromosomes were sometimes observed in diploids such as *Bellevalia saviczii* Woronow, 1927 with 2n = 8 + 1B (Gettner, 2005) and *Bellevalia koyuncui* Karabacak & Yildirim, 2015 with 2n = 8 + 2B (Karabacak et al. 2015). The occurrence of aneuploidy in a polyploid context, associated with vegetative reproduction, may indicate chromosomal changes in process providing evolutionary potential, as presumed for B-chromosomes (Johnson 2003, Weiss-Schneeweiss and Schneeweiss 2013, Bareka et al. 2015). The absence of structural differentiation and the total length of the octoploid complement, nearly twice that of the tetraploid (171.4 *versus* 93.05 µm), argue for an autopolyploidy event. Bareka et al. (2012) already concluded that autopolyploidy was the principal mechanism of polyploidization among populations occurring in Greece belonging to *Bellevalia edirnensis* hexaploid, *Bellevalia hyacinthoides* triploid and *Bellevalia ciliata* tetraploid.

### Chromosome number and polyploidy in genus *Muscari*

Karyological results on *Muscari comosum* and *Muscari maritimum* agree with previous findings on the subgenus *Leopoldia* in which species are mostly diploids (Ruiz Rejón et al. 1985, Nersesian 2001, Jafari and Maassoumi 2011, Jafari 2012a, 2012b).

All the examined specimens of *Muscari comosum* have 2n = 2x = 18 with a markedly asymmetric karyotype consisting of 2 pairs of large chromosomes and 7 pairs of small and metacentric chromosomes. Slight variations were observed in the first pair of chromosomes, sometimes viewed as telocentric (Ruiz Rejón et al. 1981, Kostovic-Vranjes 1999) or as subtelocentric (Cuñado et al. 2000, Jafari et al. 2008). Similarly, the second pair is polymorphic with submetacentric and subtelocentric chromosomes (Ruiz Rejón et al. 1985, 1990, Cuñado et al. 2000, Kostovic-Vranjes 1999).

Concerning *Muscari maritimum*, the chromosome number 2n = 18 was previously quoted by Garbari and Di Martino (1972) for specimens with unspecified origin. It is also quoted by Troìa et al. (2014) for one Tunisian population at the Cap Bon. However, in our knowledge, the karyotype structure of *Muscari maritimum* is reported here for the first time. It would be related to that of *Muscari gussonei* (Parlatore) Todaro, 1872, an endemic species to Sicily (Garbari and Di Martino 1972, Davis and Stuart 1980). The karyotype of this species consists of 10 large and 8 small chromosomes (Ruiz Rejón et al. 1985). However, karyotype of Algerian specimens collected in the Saharan border is distinguishable in having satellites located on the 1^st^ and the 5^th^ large chromosome pair.

*Muscari neglectum* belongs to the subgenus *Botryanthus* which contrasts considerably with the precedent by the occurrence of ploidy series of 2x, 3x, 4x, 5x and 6x levels (Davis and Stuart 1980, Karlén 1984, Ruiz Rejón et al. 1985, Garbari 2003, Suárez-Santiago et al. 2007). Previous chromosomal counts for *Muscari neglectum* indicate several numbers: 2n = 18, 36, 44, 54, 55, 63 and 72 (Karlén 1984). The three ploidy levels (5x, 6x, 8x) observed in Algeria confirm the extent of polyploidy in this complex. However, no diploids or tetraploids were detected in our country. In contrast, the presence of octaploid plants is significant because the 8x level was extremely rare and only few individuals having 2n = 72 were previously quoted in a population from the northern Greece (Karlén 1984). So far, only tetraploid, pentaploid and hexaploid populations of this taxon have been observed in the western Mediterranean area, precisely in the Iberian Peninsula (Ruiz Rejón et al. 1985, Suárez-Santiago et al. 2007). This is what justifies the statement generally accepted that the diploids occur only in Greece and Turkey (Karlén 1984, Garbari 2003).

## Taxonomical remarks

Morphologically, both 4x from Tiddis and 8x from Ouled Fayet and Ain Torki, are similar and belong to the endemic Bellevalia mauritanica precisely to var. eu-mauritanica Maire & Weiller, 1958. This variety is known with a geographic distribution from Central and NE Algeria throughout Tunisia and Cyrenaica. A second variety, Bellevalia mauritanica var. tunetana Battandier, 1911 is restricted to Tunisia. Concerning, the 4x population from Stidia (NW Algeria), the karyotype is distinguished by large chromosomes and satellites on the first chromosomal pair. This population of Bellevalia cf. mauritanica grows on sandy soil and differ from the type in some variable features as small scape, perigone campanulate-oblong, tepals white to sky-blue and style white. In regard to these characters and its restricted location in the NW Algeria, specimens from Stidia may be attributed to Bellevalia dubia var. variabilis (Freyn) Maire, 1941 as quoted previously (Maire 1958, Quézel and Santa 1962). However, the recent phylogenetic studies by Borzatti Von Loewenstern et al. (2013), demonstrated that *Bellevalia dubia* is diploid and narrow endemic to Sicily. Therefore, the taxonomic status of 4x samples from Stidia, considered here as Bellevalia cf. mauritanica, needs to be re-evaluated.

Within, *Muscari neglectum* group, undoubtedly the most complex within the genus *Muscari*, different authors recognize several distinct taxa based on their ploidy level. For example, Suárez-Santiago et al. (2007) on the basis of ITS sequences, argue that the pentaploid and the hexaploid Iberian populations, represent two different species, *Muscari olivetorum* Blanca, M. Ruiz Rejón & V.N. Suárez-Santiago and *Muscari baeticum* Blanca, M. Ruiz Rejón et V.N. Suárez-Santiago well separated from the tetraploid *Muscari neglectum* *s. str.* It is worth mentioning that *Muscari atlanticum* Boissier & Reuter, 1852 is the only one diploid occurring in the southern Spain and northwest of Algeria, notably at Tlemcen (Ruiz Rejón et al. 1985, Suárez-Santiago et al. 2007). Likewise, taxonomic and nomenclatural question may be raised following Maire (1958), Dobignard and Chatelain (2010-2013) and Govaerts (2015) who considered *Muscari atlanticum* as a synonym of *Muscari neglectum*. Our new reports for Tlemcen region (Mansourah) indicates only a pentaploid number 2n = 5x = 45.

In conclusion, our results contribute to a better knowledge of Hyacinthaceae in Algeria. Beside the earlier chromosomal counts, new chromosomes numbers were ascertained from Algerian populations. That is the cases of the new reports of octoploid cytotypes in *Bellevalia mauritanica* and *Muscari neglectum*. All karyological data are illustrative and reflect the east-west pattern of polyploidy at the Mediterranean scale. Further studies are needed to reconsider the taxonomic status and the evolutionary relationships of diploid and polyploid taxa in North Africa.
